# Development and Clinical Application of Left Ventricular–Arterial Coupling Non-Invasive Assessment Methods

**DOI:** 10.3390/jcdd11050141

**Published:** 2024-04-30

**Authors:** Alvaro Gamarra, Pablo Díez-Villanueva, Jorge Salamanca, Rio Aguilar, Patricia Mahía, Fernando Alfonso

**Affiliations:** 1 Cardiology Department, Hospital Universitario de la Princesa, 28006 Madrid, Spain; a.gamarralobato@gmail.com (A.G.); salamanca1982@hotmail.com (J.S.); rioaguilartorres@gmail.com (R.A.); falf@hotmail.com (F.A.); 2 Cardiology Department, Hospital Clínico San Carlos, 28040 Madrid, Spain; patmahia@gmail.com

**Keywords:** left ventricular function, ventricular–arterial coupling, elastance, non-invasive, echocardiography

## Abstract

The constant and dynamic interaction between ventricular function and arterial afterload, known as ventricular-arterial coupling, is key to understanding cardiovascular pathophysiology. Ventricular–arterial coupling has traditionally been assessed invasively as the ratio of effective arterial elastance over end-systolic elastance (E_a_/E_es_), calculated from information derived from pressure–volume loops. Over the past few decades, numerous invasive and non-invasive simplified methods to estimate the elastance ratio have been developed and applied in clinical investigation and practice. The echocardiographic assessment of left ventricular E_a_/E_es_, as proposed by Chen and colleagues, is the most widely used method, but novel echocardiographic approaches for ventricular–arterial evaluation such as left ventricle outflow acceleration, pulse-wave velocity, and the global longitudinal strain or global work index have arisen since the former was first published. Moreover, multimodal imaging or artificial intelligence also seems to be useful in this matter. This review depicts the progressive development of these methods along with their academic and clinical application. The left ventricular–arterial coupling assessment may help both identify patients at risk and tailor specific pharmacological or interventional treatments.

## 1. Introduction

**Figure 1:**
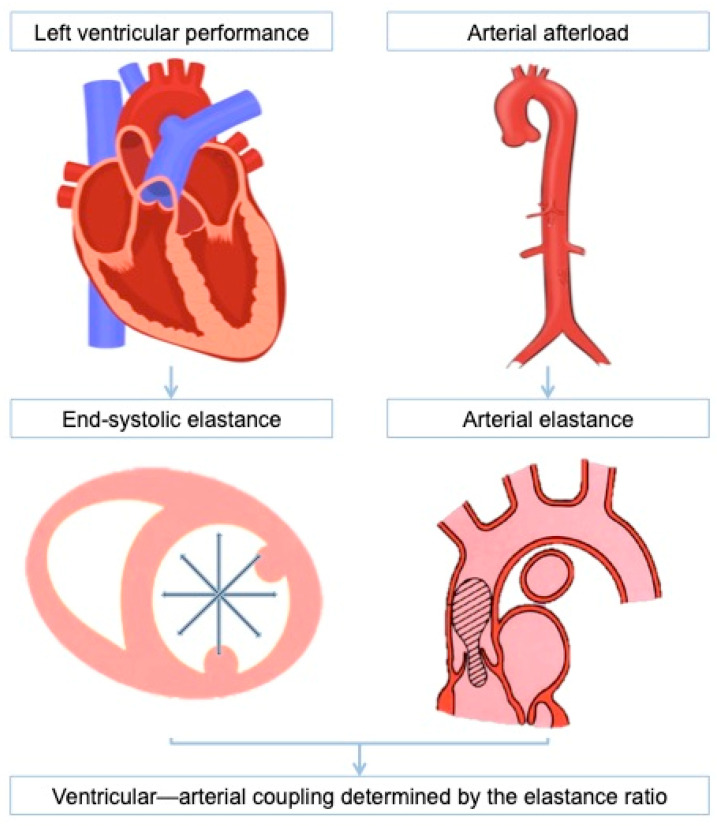
Left ventricular–-arterial coupling expressed as the elastance ratio. Arterial elastance (E_a_) is calculated as end-systolic pressure over stroke volume. End-systolic elastance (E_es_) is calculated as ventricular end-systolic pressure over ventricular end-systolic volume.

The concept of ventricular–arterial coupling (VAC) was first developed fifty years ago, aiming to integrate into a single system of two structures that are deeply connected both anatomically and functionally: the heart and the arteries [1,2,3,4] (Figure 1). In order to analyze their relationship, both components need to be represented mathematically with the same magnitude and elastance, which measures changes in pressure for each unit change in volume (unit: mmHg/mL).

**Figure 2:**
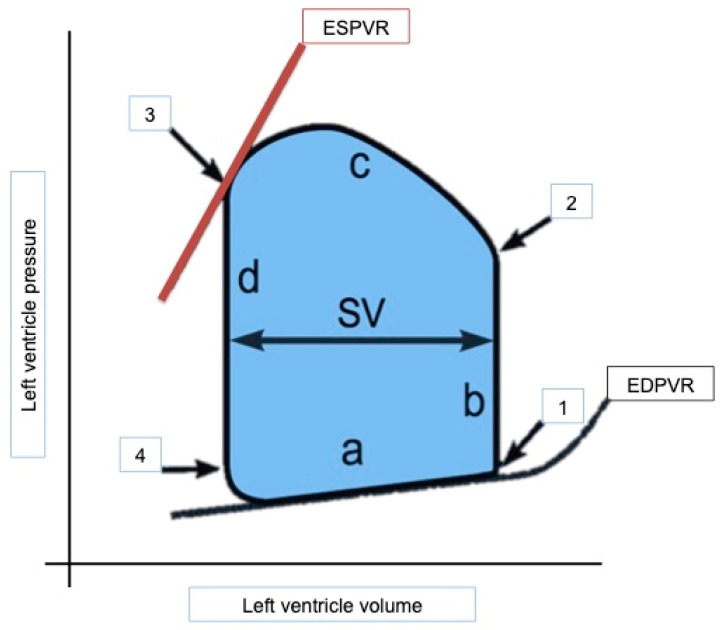
Pressure–volume loop representing the relationship between intraventricular pressure (ordinate) and volume (abscissa) measured simultaneously throughout the cardiac cycle (a: diastole; b: isovolumetric contraction; c: systole; d: isovolumetric relaxation; 1: mitral valve closure; 2: aortic valve opening; 3: aortic valve closure; and 4: mitral valve opening). SV: stroke volume; ESPVR: end-systolic pressure–volume ratio; EDVPR: end-dyastolic pressure–volume ratio.

The study of VAC initially required an invasive approach and the use of high-fidelity conductance microcatheters to calculate sets of pressure–volume (PV) loops for different preload conditions in a given patient. PV loops represent the relationship between volume and pressure, measured simultaneously throughout the cardiac cycle (diastole-isovolumetric contraction-systole-isovolumetric relaxation; Figure 2). As well as calculating VAC, PV loops allow the study of ventricular stroke work [5] and ventricular efficiency, which are linearly related to myocardial oxygen consumption (MVO2) in canine heart models [6,7].

The performance and stiffness of the ventricular chamber, defined as end-systolic elastance (E_es_), is determined by the ratio of ventricular end-systolic pressure over ventricular end-systolic volume. It is relatively load-independent [1,2,3,4], losing linearity in extreme conditions. It is sensitive to chamber remodeling and stiffening [8], as well as to contractility and inotropic modulation [9]. It is represented graphically by the end-systolic pressure–volume ratio (ESPVR) as the slope of the line formed when connecting the different end-systolic pressure–volume points of the pressure–volume loops for different preload conditions in a given patient. At rest, its value is 2.3 ± 1.0 mmHg/mL [10].

On the other hand, the afterload that opposes the heartbeat, known as effective arterial elastance (E_a_), is calculated as the division between end-systolic pressure and stroke volume and has a resting value of 2.2 ± 0.8 mmHg/mL [10]. It is not only influenced by static components such as peripheral resistances, but by pulsatile components as well, such as aortic impedance, reflection waves, or heart rate [11].

Thus, VAC is defined as the ratio of E_a_ over E_es_ and helps understand the pumping capacity of the heart in relation to the load and adaptability with which the arterial system opposes it.

In order to overcome the need for invasive approaches, several non-invasive methods have been developed for estimating both E_es_ and E_a_ in recent years. The objective of this review is to analyze the development and applicability in routine clinical practice of simplified echocardiographic methods for calculating left VAC, their evidence in different pathologies, as well as the possibility for VAC-directed treatment.

## 2. Estimation of Left End-Systolic Elastance (Figure 3)

**Figure 3:**
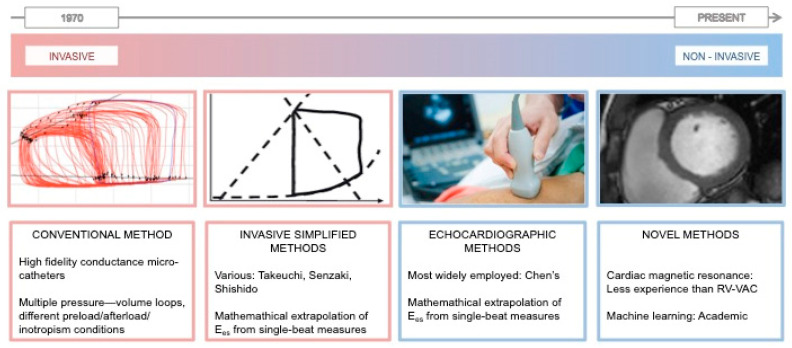
Chronological development of conventional, simplified, and non-invasive methods for the estimation of left end-systolic elastance.

### 2.1. Simplified Single-Beat Invasive Methods

Since E_es_ is calculated as the slope of the end-systolic pressure–volume relationship (ESPVR), only two end-systolic pressure–volume points should be needed to draw this line. The objective of several authors was to simplify this calculation using information from a single pressure–volume loop (Table 1).

- Takeuchi and Colleagues [12] described the Simulated Isovolumetric Pressure curves method, which estimates the maximum pressure (P_maxE_) for a given end-diastolic volume by fitting an isovolumetric pressure curve on the invasive pressure record of an ejection cycle. The slope determined by this estimated maximum pressure point and tangential to the measured end-systolic pressure–volume point corresponds to the estimated E_es_ (E_esE_).

E_esE_ and P_maxE_ were calculated for three preload situations at the baseline, under vasodilator and vasopressor treatment, and compared with the values calculated using the conventional method. The E_es_ values measured by the conventional method (mean E_es_ = 4.9 ± 2.7 mmHg/mL/m^2^) were similar to those estimated (5.0 ± 2.2 mmHg/mL/m^2^), presenting a good correlation r = 0.91, *p* < 0.001, and the results were reproducible for different loading situations.

- Senzaki and colleagues [13] developed the normalized elastance method by collecting information from 200 equidistant points of a total of 72 PV loops chosen randomly from the total recorded in 52 individuals. A normalized time-varying elastance curve was calculated with its corresponding pressure and volume values for each moment (t). Thus, in a specific patient, after recording a PV loop, E_es(SB)_ could be calculated from the following equations after calculating V_0_.

E_es(SB)_ = P_(tMax)_/[V_(tMax)_ − V_0(SB)_] (1)

V_0(SB)_ = [P_N(tN)_V_(tMax)_ − V_(tN)_E_N(tN)_]/P_N(tN)_ − E_N(tN)_ (2)

where tMax is the time to reach the end-systolic pressure–volume point and tN seems to work better at values between 0.25 and 0.35 s.

Conventional and simplified E_es_ and V_0_ estimation demonstrated a very good correlation at the baseline and under different preload and inotropic situations (r = 0.92, *p* < 0.0001).

- Shishido and colleagues [14] used a slightly different approach, not based on volumetric parameters, and described the bilinearly approximated elastance method. They simplified the ventricular elastance equation by Suga and Sagawa [1,2].

E(t) = P(t)/[V(t) − V0](3)

by approximating two straight lines, one for the isovolumetric contraction phase and another for the ejection phase. The relation between the slopes of these lines is the variable α. This is taken into account as, for a given volume, the elastance is proportional to the pressure according to formula,

E_es_/E_a_ = (P_max_ − P_es_)/P_es_ (4)

and after substituting P_max_,

E_es_/E_a_ = P_ad_/P_es_ (1 + α × ET/PEP) − 1 (5)

The equations for the corresponding approximation lines can be expressed as ratios of elastance or pressure per time, allowing the variable α to be calculated as follows:

α = [(E_es_ − E_ad_)/ET]/(E_ad_/PEP) = [(P_es_ − P_ad_)/P_ad_] × (PEP/ET) (6)

Here, the times _es_ and _ad_ correspond to the end of the systole and the aortic valve opening (arterial diastole), respectively. The pre-ejection period (PEP) corresponds to the time of isovolumetric contraction (from the beginning of the contraction, the moment in which the dP/dT reaches 10% of the maximum, until the beginning of the rise in the aortic pressure curve). Ejection time (ET) corresponds to the duration of the ejective phase.

After comparing conventional and novel measures of E_es_/E_a_ values, a good correlation was observed (r = 0.925, *p* < 0.05).

The main limitations of these invasive simplified methods are related to the non-linearity of the ESPVR in extreme load situations or the assumption of a constant V_0_ throughout the entire cardiac cycle [13]. However, again, in a canine model, Little and colleagues concluded that an extreme preload reduction (bicaval occlusion) does not limit the estimation of E_es_ [15].

Reproducibility after loading intervention was studied by preload reduction in all methods except for Takeuchi’s. This nuance was found to be significant in Wo’s comparison of single-beat methods [16] when the loading intervention was made, both with preload reduction and an afterload increase. Sishido’s method yielded the strongest correlation for the two different loading interventions.

Reproducibility for different inotropic states was not always studied in depth [12,14], and the results were more frequently erroneous in patients with severe left ventricular systolic dysfunction [12,14], corresponding with severe VA uncoupling.

### 2.2. Echocardiography-Based Non-Invasive Methods

Once more, through complex mathematical calculations, these methods allow E_es_ to be extrapolated from the information obtained in a single cardiac cycle, avoiding the need for an invasive approach or for the characterization of multiple PV loops.

- Chen and colleagues [17] developed a method that is considered to be the non-invasive gold standard for E_es_ estimation. They compared invasive, conventional measures, which were estimated non-invasively in 50 individuals: 7 were healthy while 13 patients underwent coronary angiography without obstructive coronary artery disease (CAD) or ventricular dysfunction, 13 patients had CAD, 8 patients had hypertensive heart disease, 5 patients had dilated cardiomyopathy (DCM), 1 patient had hypertrophic cardiomyopathy (HCM), 1 patient had constrictive pericarditis, and 2 were heart transplant patients.

**Figure 4:**
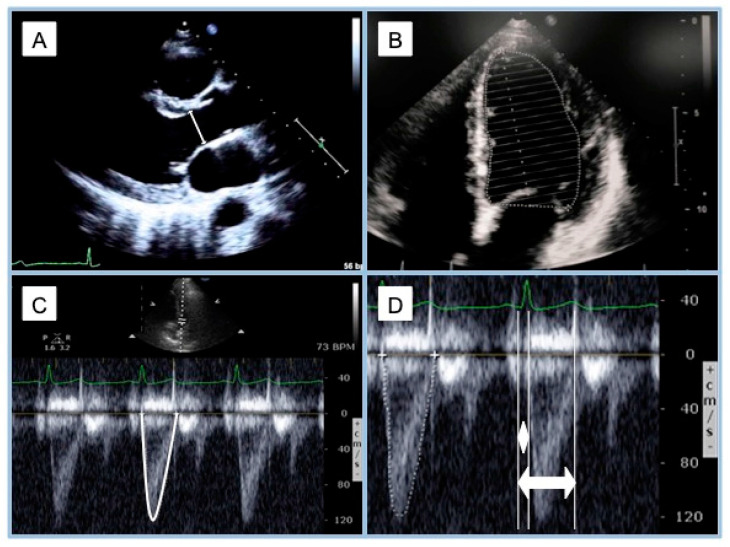
Echocardiographic estimation of left end-systolic elastance using Chen’s method. (**A**): Parasternal long-axis view, left ventricle outflow tract diameter measure; (**B**): left ventricle ejection fraction measured by Simpson’s biplane method, apical 4-chamber view in this picture; (**C**): apical 5-chamber view, the velocity–time integral of left ventricle outflow tract pulsed-wave Doppler; (**D**): apical 5-chamber view, left ventricle outflow tract pulsed-wave Doppler, pre-ejection time (white diamond) and total ejection time (white double arrow).

In order to estimate E_es_ in this method, it was necessary to calculate the stroke volume (SV) by measuring the velocity–time integral (VTI) in the LV outflow tract (LVOT) in the 5-chamber apical view as well as the LVOT area calculated from its diameter in the parasternal long-axis view (Figure 4). It is also necessary to calculate the ejection fraction (EF) and measure the (BP) at two different moments of the heart cycle (P_d_ at the beginning of ejection and P_es_ at the end of the systole).

While VAC coupling measured by the elastance ratio is inversely related to EF, and therefore, E_es_ is directly proportional to EF, the latter might be an oversimplified marker of ventricular performance in many scenarios [18,19].

The algorithm for estimating E_es(SB)_ is based on the Suga and Sagawa [1,2] equation used for previous models and assumes a constant value of V0. By applying this equation to different moments of the cycle, such as end-systolic (t_es_) or the beginning of ejection (t_D_) and substituting factors, the following equation is obtained:

E_es(SB)_ = P_(tMax)_ ∕ [V_(tMax)_/V_0(SB)_] (7)

in which the estimated normalized elastance at the time of onset of ejection (E_Nd(est)_) is calculated from data obtained from another independent cohort of 23 subjects in whom the conventional method of measurement was carried out, developing the following equation:

E_Nd(est)_ = 0.0275 − 0.165 × EF + 0.3656 × (P_d_/P_es_) + 0.515 × E_Nd(avg)_ (8)

in which E_Nd(avg)_ is given by the polynomial function

E_Nd(avg)_ = Σ_i = 0_ a_i_ × t_Nd_^i^ (9)

and in which a_i_ is equivalent to 0.35695, −7.2266, 74.249, −307.39, 684.54, −856.92, 571.95, and −159.1 for values from I = 0 to 7, respectively. The value of t_Nd_ is equivalent to the ratio between the pre-ejection time and the total ejection time, both referenced with the R wave on the electrocardiogram (EKG).

When comparing the new non-invasive method with the conventional technique, a good correlation was observed between E_Nd(est)_ and invasive E_Nd_ (r = 0.88, *p* < 0.00001), as well as between the estimated E_es(SB)_ and the invasive E_es_ with the following regression equation:

E_es_ = 0.78 × E_es_(sb) + 0.55 (r = 0.81, SEE = 0.50, *p* < 0.0001). (10)

The mean difference between E_es_ and E_es_(SB) was 0.03 mmHg/mL, with 80% of the erroneous estimates below 0.6 mmHg/mL. In this sense, since normal resting values for E_es_ are around 2 mmHg/mL, <1 mmHg/mL in dilated and dysfunctional ventricles, and around 4 mmHg/mL in hypertrophic ventricles, the discriminative capacity of this technique does not seem to be compromised. This good correlation was also patent when comparing the new and conventional methods after load intervention^16^ and under dobutamine stimulation [17].

Thus, the work of Chen and colleagues provided the first completely non-invasive validated model for the estimation of E_es_ using information easily accessible through a sphygmomanometer, an EKG, and pulsed-Doppler echocardiography.

A limitation of this model seems to be its intra-individual reproducibility, given that the measurement was repeated monthly for 3 months in 7 subjects, showing an average coefficient of variation of 20 ± 6%, mainly in relation to the change in stroke volume.

Subsequent research to characterize E_es_ using Chen’s method in elderly patients indicated significantly higher values, which were more pronounced in women, similar to those in young people with hypertensive heart disease [20,21]. Parallel to E_es_, there was also an age-related increase in E_a_ secondary to arterial stiffening that propitiates the elastance ratio to remain relatively unchanged in the elderly [8,21]. Nevertheless, the E_a_/E_es_ ratio remains unchanged due to higher values of both E_a_ and E_es_, conditioning a higher systolic pressure sensitivity to cardiac preload [8,19].

However, the main limitation of the application in daily clinical practice of the model proposed by Chen et al. is the complexity of mathematical calculations. To overcome this obstacle, an editorial in the European Journal of Heart Failure [22] was recently published, showing the steps for the correct measurement of E_es(SB)_ in clinical practice. Links to Excel© or iElastance© spreadsheets in which to enter data and automate calculations are also provided.

- Bauer and colleagues [23] proposed using the systolic acceleration in LVOT (LVOT_Acc_) as a surrogate of E_es_ measured invasively based on the results obtained from an ovine model with 18 sheep (4 healthy, 6 with aortic regurgitation and 8 with myocardial infarction of the first diagonal artery).

LVOT_Acc_ was calculated using pulsed-Doppler as the ratio between the average of three consecutive recordings of the peak velocity (pVel) and the time to reach the peak velocity (t-pVel).

When comparing E_es_ and LVOT_Acc_ in specific scenarios, they observed that the increase or reduction in preload and afterload (blood, angiotensin, or nitroprusside infusion) did not significantly alter either measure (one-way ANOVA, *p* = 0.06). However, acute ischemia following occlusion of the proximal anterior descending or circumflex arteries significantly reduced both parameters (one-way ANOVA, *p* = 0.002).

A strong linear correlation was found between E_es_ and LVOT_Acc_, as expressed by the following continuity equation:

E_es_ = 0.78 × E_es_(sb) + 0.55 (r = 0.81, SEE = 0.50, *p* < 0.0001) (11)

which did not change when correcting the pVel and t-pVel values by heart rate.

The findings of this study in an animal model are promising, given the simplicity of the measures and the ability to predict clinical changes, such as ischemia. Nevertheless, despite the LVOT_Acc_ measure presenting a good intraobserver correlation, interobserver differences of up to 16.8% were reported, mainly related to the difficulty in identifying the beginning of the acceleration curve in LVOT, as well as the point of maximum velocity. Another important limitation is the turbulence produced by valvular or subvalvular obstruction. Furthermore, the variability of LVOT velocity depending on age could limit the application of this model in the elderly.

- In a slightly different line of work, echocardiographic reference ranges were recently published for non-invasive myocardial work indices in healthy volunteers [24]. In this study, authors seek to define normal values for global work index (GWI), global work waste (GWW), and global work efficiency (GWE) in an attempt to incorporate systolic function, myocardial deformation, and arterial load in the so-called pressure–strain loops and avoid the influence of afterload over strain echocardiography [25].

### 2.3. Multimodal Imaging-Based Non-Invasive Methods

The development of new technology applied to the study of the cardiovascular system has allowed the adaptation of techniques such as cardiac magnetic resonance (CMR) to the study of VAC. In this field, experience is greatest regarding the right ventricle and its relationship with pulmonary circulation, although in recent years, models have also been described for the non-invasive estimation of left E_es_ using CMR.

Seemann and colleagues [26] adapted the time-dependent elastance model expressed according to the equation proposed by Stergiopulos [27]; this was optimized through information obtained from the invasive characterization of 875 porcine-model studies of PV loops.

The correlation between the values measured invasively and by CMR in a porcine model was good, especially for stroke work (intraclass correlation coefficient = 0.93; bias, −0.02 ± 0.03 J). Subsequent validation was carried out in humans, comparing volunteers without heart disease and patients with heart failure and reduced EF (HFrEF) (14 patients with ischemic heart disease and 14 patients with DCM). The method under study was able to characterize and discriminate both populations. In a subsequent study [28], the same group adapted this method to avoid the loss of precision at high heart rates.

### 2.4. Artificial Intelligence and Machine Learning to Help Estimate End-Systolic Elastance

The growing accessibility to supercomputers has favored the development of other non-invasive models for the estimation of E_es_. These deep learning techniques are able to mathematically model the cardiovascular system and estimate the value of E_es_ based on simple information obtained through echocardiography or the use of a sphygmomanometer.

For example, in a recent study by Pagoulatou and colleagues [29], the authors matched the one-dimensional model of the arterial tree, divided into 103 segments, and a Navier–Stokes equation was solved for each one in Windkessel’s three-element model. After adapting the model to the age, height and heart rate of the subject, they introduce the values of systolic and diastolic blood pressure (SBP and DBP), LVOT diameter and VTI value with which E_es_ could be estimated non-invasively with a good degree of correlation versus the conventional measurement in 10 patients with heart failure and preserved EF (HFpEF) and another 9 healthy subjects (normalized root mean square error = 9%, ρ = 0.89, bias = −0.1 mmHg/mL, and limits of agreement = [−0.9, 0.6] mmHg/mL).

Other authors investigated the possibility of estimating E_es_ through convolutional neural networks (CNNs) by analyzing the morphology of the BP wave at the brachial level [30] or through artificial intelligence (Extreme Gradient Boosting, XGBoost), by analyzing the ejection and pre-ejection times [31]. The results of these studies are promising, but they are based on computer simulations and have not been validated in clinical models. On the other hand, they require very advanced and expensive computer equipment, which is not available in most work environments.

## 3. Non-Invasive Estimation of Arterial Elastance

In routine clinical practice, E_a_ is most frequently estimated by calculating the relation between end-systolic BP and SV. The latter is calculated by pulsed-Doppler echocardiography, while end-systolic BP can be estimated as 90% of the SBP measured at the brachial level with a sphygmomanometer and a linear relationship with the slope 1.01, where r = 0.7525 and *p* < 0.0001 [32,33].

Different authors propose using other BP measurements to estimate E_a_. For example, in a recent study on the porcine model, Monge and colleagues [34] proposed the ratio between mean arterial pressure (MAP) and SV as the best non-invasive and accessible surrogate of E_a_, above SBPx0.9 or the dicrotic wave pressure. They based this recommendation on its better correlation with E_a_ calculated invasively in different preload and postload situations and on the consistency of the values measured with a sphygmomanometer at the brachial or femoral level.

Authors like Chemla and Teboul defend the invasive characterization of E_a_, although they consider the non-invasive approach using SBPx0.9 to be more reliable than the use of MAP [35].

When assessing VAC in critical patients, in whom the invasive measure of BP is routinary, it is worth taking into consideration that pulse wave morphology and invasive BP values may be synchronized with EKG and displayed on the screen of many modern portable ultrasonographers, thus facilitating a simultaneous measure of end-systolic invasive pressure and echocardiographic LV volume.

## 4. Left Ventricular–Arterial Coupling in Different Clinical Scenarios

Since the first invasive methods were developed to construct and analyze PV loops, many studies have assessed the prognostic information behind E_a_/E_es_ values and have speculated on the possibility of VAC-directed therapies.

Before addressing specific pathologies, it is worth remembering the work of Asanoi and colleagues [36], who, in 1989, differentiated the distinctive patterns for healthy patients, patients with left ventricular EF (LVEF) 40–50%, and patients with LVEF < 40%; and their corresponding mechanoenergetic correlate.

In the first group, the E_a_/E_es_ ratio ranged around 0.5–0.7, which represented maximum mechanical efficiency. In contrast, in the last group, E_es_ values tended to be half that of E_a_, obtaining an E_a_/E_es_ ratio greater than two, resulting in a population very susceptible to variations in E_a_.

**Figure 5:**
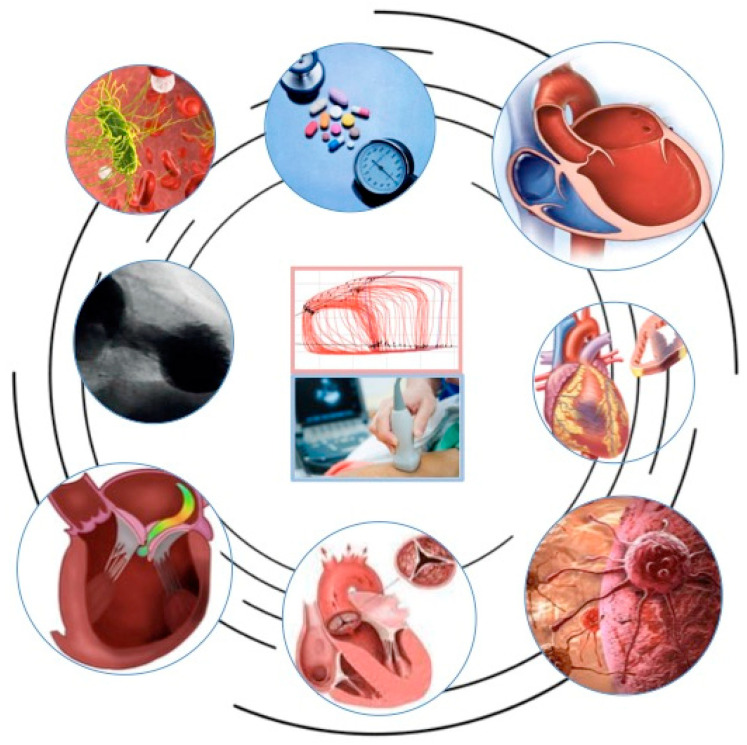
Left ventricular–arterial coupling in different clinical scenarios: hypertension, heart failure, coronary artery disease, cardio-oncology, aortic valve stenosis, mitral regurgitation, Takotsubo syndrome, or septic shock.

Thus, it is easy to understand the therapeutic simplification proposed by Monge [37] and Little [38] in their reviews on VAC and its clinical application in critically ill patients where an imbalance between the left ventricle and the arterial system, with a ratio E_a_/E_es_ > 1, might be due to an increase in afterload or a decrease in contractility. In the first case, e.g., hypertensive crisis, it is reasonable to start vasodilator treatment to reduce E_a_ and allow balance to be restored. On the other hand, in the second case, e.g., of severe systolic dysfunction, inotropic support could be started to improve contractility. This simplified example can be adapted to other clinical scenarios in critical or stable patients (Figure 5).

### 4.1. Hypertension, Diabetes, and Chronic Heart Failure with Preserved Ejection Fraction

Different studies have highlighted the influence of hypertension [39] and diabetes [32] over VAC through ventricular and arterial stiffening, leading to a disbalanced E_a_/E_es_ similar to that found in patients with HFpEF. In fact, hypertension and diabetes are two of the main risk factors for developing HfpEF [40], along with other cardiovascular diseases. In these patients, E_a_/E_es_ may be normal due to a parallel increase in both factors, resulting in abnormally high values for both E_a_ and E_es_.

Antihypertensive treatment has been shown to improve VAC, LV systolic, and diastolic function and reduce LV hypertrophy [41] in various studies as extensively described by Ikonomidis and colleagues [42] in a consensus document for the European Society of Cardiology. Treatment with angiotensin-converting enzyme inhibitors (ACE-i), angiotensin-II receptor blockers (ARB), and dihydropyridine calcium antagonists yielded the best results [41,43]. Not only pharmacotherapy but also a low-sodium diet has been shown to improve VAC measured by Chen’s method [44].

The spectrum of patients with HFpEF is very broad, so apart from the already mentioned influence of hypertension and diabetes, it is also worth mentioning the influence of inflammatory conditions on LV function and remodeling. Conditions such as rheumatoid arthritis, lupus, ankylosing spondylitis, psoriasis, gout, and medium- and large-vessel vasculitides may accelerate arterial and myocardial stiffening [40,42], while anti-inflammatory treatment with Anakinra or Tocilizumab has been shown to improve VAC measures, such as systemic arterial compliance or pulse-wave velocity, respectively, but not E_a_/E_es_ [40].

### 4.2. Chronic Heart Failure with Reduced Ejection Fraction

Along with the New York Heart Association (NYHA) functional class, increased natriuretic peptides, reduced LVEF or longitudinal global strain, E_a_/E_es_ is strongly correlated with adverse clinical outcomes [45].

Multiple studies have shown that the administration of certain drugs (e.g., Sacubitril-Valsartan, Carvedilol, or Spironolactone) improves the E_a_/E_es_ ratio, and thus, VAC in addition to their known clinical benefits [46,47,48,49].

The initiation of cardiac resynchronization therapy has also shown a significant immediate reduction in the E_a_/E_es_ ratio (measured by Chen’s method), related to an increase in LVEF and a reduction in interventricular dyssynchrony [50].

Similarly, in patients with LVEF < 45% included in a 20-session cardiac rehabilitation program, an improvement in the E_a_/E_es_ ratio (measured by Chen’s method) was demonstrated, correlated with an improvement in ventricular mechanical efficiency, although no correlation was demonstrated with an improvement in peak O_2_ consumption measured by cardiopulmonary exercise testing [51].

### 4.3. Coronary Artery Disease

E_a_/E_es_ measured by Chen’s method had an independent prognostic value, similar to BNP, in patients with previous myocardial infarction [52]. In this same study, an E_a_/E_es_ ratio lower than 1.47 conferred a lower mortality.

An impaired VAC reserve (E_a_/E_es_ change between stress and rest) was able to identify patients at risk of heart failure-related events amongst patients with known or suspected CAD and negative stress echocardiography [53].

Regarding the controversial management of stable ischemic heart disease, a recent study demonstrated that percutaneous revascularization led to a significant improvement in the E_a_/E_es_ ratio (measured by Chen’s method, due to improved E_es_ after a 6-month follow-up [54].

### 4.4. Cardio-Oncology

In recent years, the importance of early detection of patients developing chemotherapy-related myocardial dysfunction has been highlighted, emphasizing the importance of detecting patients at risk of toxicity. On this matter, the E_a_/E_es_ ratio (measured by Chen’s method) has shown good capacity for both, identifying patients at risk or with established toxicity to anthracyclines/trastuzumab [55,56,57], whether this toxicity is due to the affectation of contractility (decreased E_es_) or vascular resistance (increased E_a_), which appears to improve during follow-up [58].

### 4.5. Aortic Valve Stenosis

For the study of aortic stenosis, the time-varying elastance model must be adapted to the fact that there is an obstruction between the ventricular cavity and the aorta. A specific index called valvulo–arterial impedance (Zva) was proposed by Briand and colleagues [59] to combine vascular and valvular factors opposing LV blood ejection. This index represents the pressure needed to eject 1 mL of blood and allows the prediction of events in asymptomatic patients with severe aortic stenosis. It is calculated as follows:

Zva  =  LV pressure/SVi (mL/m^2^/mmHg) (12)

where LV pressure is the sum of SBP and the mean pressure aortic gradient; SVi is the stroke volume indexed to the body surface.

In a recently published article, Migliore and colleagues [60] correlated the evolution of E_a_ and E_es_ measured according to Senzaki’s method, throughout the four stages of cardiac involvement in aortic stenosis previously proposed by Généreux and colleagues [61]. A progressive increase in E_a_ was seen accompanied by a decrease in E_es_ in stage 4, which produced an increase in the E_a_/E_es_ ratio.

In previous studies, an improvement in hemodynamic parameters measured invasively and non-invasively was already observed immediately after percutaneous aortic valve replacement [62].

### 4.6. Mitral Valve Regurgitation

One of the main fears before intervening in a patient with severe mitral regurgitation is the response of the LV and its hypothetical deterioration after limiting its retrograde escape route. In 18 patients, MitraClip implantation reduced regurgitant volume, slightly reduced LVEF, and increased stroke volume by 30% but did not modify the E_a_/E_es_ ratio measured by Chen’s method [63].

A larger observational study demonstrated a reduction in end-systolic and end-diastolic volume, accompanied by an improvement in LVEF, stroke volume, and VAC (measured by Chen’s method) for patients with mitral regurgitation and LVEF > 40%, regardless of whether the etiology was degenerative or functional [64]. In this same study, patients with LVEF < 40% improved stroke volume and LVEF without improving the E_a_/E_es_ ratio. The clinical benefit in terms of functional class improvement was similar for both groups.

### 4.7. Takotsubo Syndrome

Takotsubo syndrome (TTS) is an increasingly recognized syndrome with a distinctive phenotype characterized by a form of transient regional wall motion abnormalities in the absence of culprit epicardial coronary artery disease on angiography. The study of PV relations may provide in-depth information regarding VAC and cardiac energetics and efficiency in this fascinating and elusive disease. In one study by Medeiros and colleagues [65], LV in TTS and acute myocardial infarction (AMI) patients were retrospectively analyzed. Compared to the controls, both TTS and AMI patients exhibited higher LV volumes, diastolic pressures, and diastolic stiffness. Additionally, indexes of contractility and ventricular–arterial coupling were similarly abnormal in both TTS and AMI groups. Furthermore, recently, Stiermaier and colleagues [66] recorded left ventricular PV loops in TTS patients and compared the results with healthy controls. TTS patients consistently showed impaired parameters of LV contractility beyond EF, including E_es_, end-systolic volume at 150 mmHg, and dP/dtmax, indicating reduced cardiac contractility. The LV PV diagram exhibited a rightward shift, with increased LV end-diastolic and end-systolic volumes but preserved stroke volume. Diastolic function showed prolonged active relaxation, while diastolic stiffness was preserved. The mechanical efficiency of the LV was significantly reduced in TTS, characterized by reduced stroke work and increased potential energy, indicating inefficient myocardial energetics.

Summarily, during the acute phase of TTS, hemodynamic changes are marked by reduced cardiac contractility, inefficient myocardial energetics, and prolonged active myocardial relaxation, while diastolic passive stiffness remains unaltered [67]. Information on the non-invasive evaluation of VAC in TTS is scarce, and whether a non-invasive parameter could help avoid an invasive angiography is merely hypothetical. Much investigation is needed in this promising field of study.

### 4.8. Septic Shock

Guarracino and colleagues [33,68] demonstrated that patients with septic shock present not only a reduction in E_a_ but also a relatively greater reduction in E_es_, which conditions an unbalanced E_a_/E_es_ ratio of around 1.8. In these patients, the administration of fluid therapy with or without dobutamine was superior to the use of norepinephrine for normalizing the E_a_/E_es_ ratio, yet no clinical outcomes were reported.

### 4.9. Very Elderly

As previously mentioned, the elastance ratio did not seem to vary significantly with age, secondary to a parallel increase in both E_a_ and E_es_ [8,21]. However, there is a growing population of very elderly patients in routine clinical practice, underrepresented in previous studies, for whom the increase in E_es_ is disproportionate, especially in women, leading to a lower elastance ratio, which should be considered when addressing this issue in such patients [69].

## 5. Conclusions

For decades, the study of ventricular–arterial coupling has been fundamental to improving the pathophysiological understanding of cardiovascular diseases. The invasive characterization of pressure–volume loops has been fundamental for the development of pharmacological therapies as a variable of prognostic interest or even as a surrogate objective in research work.

The information provided by conventional and invasive methods has been as abundant as numerous attempts to develop non-invasive methods for the estimation of pressure–volume loops. These methods allow obtaining as much detailed information as possible through simple low-risk approaches, thanks to mathematical formulas that reproduce the behavior of the cardiovascular system. There are, however, inherent limitations to the mathematical estimation of physiological processes.

Among these methods, the one developed by Chen and colleagues stands out due to its correlation with invasive measures and its application in clinical practice and multiple research protocols. However, efforts to develop novel methods continue, both through advanced imaging techniques and computing, artificial intelligence, and machine learning.

Adapting these methods to daily clinical practice could bring the clinician closer to dynamically understanding the cardiovascular physiology of each specific patient and, perhaps, facilitate the task of tailoring individualized pharmacological or interventional treatments.

## Figures and Tables

**Table 1:** Simplified single-beat invasive methods for the estimation of left end-systolic elastance.

Simplified Single-Beat Invasive Methods			
Simulated isovolumetric pressure curves methodTakeuchi et al. 1991 [12]	Human model	Reproducible under different preload, afterload and inotropism conditions	r = 0.91, *p* < 0.001
Normalized elastance methodSenzaki et al. 1996 [13]	Human model	Reproducible under different preload and inotropism conditions	r = 0.92, *p* < 0.0001
Bilinearly aproximated elastance methodShishido et al. 2000 [14]	Canine model	Reproducible under different preload and inotropism conditions	r = 0.925, *p* < 0.05
